# Clinical Validation of a Serum Protein Panel (FLNA, FLNB and KRT19) for Diagnosis of Prostate Cancer

**DOI:** 10.4172/2155-9929.1000323

**Published:** 2017-02-08

**Authors:** Shobha Ravipaty, Wenfang Wu, Aditee Dalvi, Nikunj Tanna, Joe Andreazi, Tracey Friss, Allison Klotz, Chenchen Liao, Jeonifer Garren, Sally Schofield, Eleftherios P Diamandis, Eric A Klein, Albert Dobi, Shiv Srivastava, Poornima Tekumalla, Michael A Kiebish, Vivek Vishnudas, Ranga Prasad Sarangarajan, Niven R Narain, Viatcheslav R Akmaev

**Affiliations:** 1 Berg, LLC, 500 Old Connecticut Path Framingham, MA 01701, USA; 2 Department of Pathology and Laboratory Medicine, Mount Sinai Hospital, Toronto, Ontario, Canada; 3 Glickman Urological and Kidney Institute, Cleveland Clinic, Cleveland, USA; 4 Department of Surgery, Center for Prostate Disease Research, Uniformed Services University of the Health Sciences and the Walter Reed Military Medical Center, Bethesda, Maryland, USA

**Keywords:** Prostate cancer, Prostate specific antigen, Biomarker, FLNA, FLNB, KRT19

## Abstract

This study reports on the development of a novel serum protein panel of three prostate cancer biomarkers, Filamin A, Filamin B and Keratin-19 (FLNA, FLNB and KRT19) using multivariate models for disease screening and prognosis. ELISA and IPMRM (LC-MS/MS) based assays were developed and analytically validated by quantitative measurements of the biomarkers in serum. Retrospectively collected and clinically annotated serum samples with PSA values and Gleason scores were analyzed from subjects who underwent prostate biopsy, and showed no evidence of cancer with or without indication of prostatic hyperplasia, or had a definitive pathology diagnosis of prostatic adenocarcinoma. Probit linear regression models were used to combine the analytes into score functions to address the following clinical questions: does the biomarker test augment PSA for population screening? Can aggressive disease be differentiated from lower risk disease, and can the panel discriminate between prostate cancer and benign prostate hyperplasia? Modelling of the data showed that the new prostate biomarkers and PSA in combination were better than PSA alone in identifying prostate cancer, improved the prediction of high and low risk disease, and improved prediction of cancer versus benign prostate hyperplasia.

## Introduction

Prostate cancer (PrCa) is a leading cause of cancer deaths in American men [1,2]. Currently the most prevalent method for detection of PrCa includes screening with a prostate-specific antigen (PSA) blood test followed by a digital rectal examination and diagnostic prostate biopsy. The PSA-based test is first-line for screening of PrCa [3,4]. However, in 2012 the USPTF issued a recommendation against use of PSA-based screening due to its limitations in accuracy. Use of this test has resulted in over-diagnosis and overtreatment of nonlethal cancers, resulting in reduced quality of life of patients who did not require treatment [4]. Thus, there is a need for a diagnostic test with increased predictive power which may reduce the frequency of biopsy, over detection and overtreatment of the 40% to 50% of current cases that are indolent [3].

Recent advances in PrCa detection include: tests that measure variants of PSA in blood [5], expression or hypermethylation of PrCa specific genes [6], or proteins in biopsy tissues [7], and expression of PrCa associated genes [8], or fusion genes in post-digital rectal examination urine [9], or urine exosomes [10]. Markers such as the *TMPRSS2-ERG* fusion and *PCA3* have improved diagnostic accuracy, although discrimination between low risk and more aggressive disease states remains challenging. Indeed, there is a continued need for core biopsy specimens to confirm diagnosis [3].

Therefore, improvements in identification of high-risk cancers from low risk cancers need to be developed when diagnosing prostate cancer, in order to administer the appropriate course of action [10]. High-risk prostate cancers (with a Gleason score of at least 7) have been shown to be more accurately identified using the STHLM3 model, which suggests that structured screening that includes clinical factors, PSA and some PSA derivatives, and germline allelic variants, could reduce the number of prostate biopsies by about a third, when compared to PSA alone [11]. Similarly, a study in 2012 showed that a blood-based Biomarker Panel (CRTAM, CXCR3, FCRL3, KIAA1143, KLF12, TMEM204) could identify men with aggressive prostate cancer, thereby reducing the over-diagnosis and overtreatment that currently results from using PSA alone [12]. These and other studies demonstrate the improvements that have been made in predicting aggressive cancers. Nevertheless, there remains a need for better stratification of patients to low and high-risk forms of the disease.

This study utilizes a novel panel of serum biomarkers to augment the diagnosis of prostate cancer in conjunction with the PSA test. The biomarkers described herein are novel entities and are not PSA derivatives. The panel was developed by using a multiomic approach that defined filamin-A (FLNA), filamin-B (FLNB), and keratin-19 (KRT19) in the panel [13]. New ELISAs for FLNA and FLNB were developed along with immunoprecipitation multiple reaction monitoring (IPMRM) for FLNA, which resulted in significant improvements in the context of PrCa detection and prognosis.

## Materials and Methods

### Human and animal rights

All procedures performed in studies involving human participants were in accordance with the ethical standards of the institutional and/or national research committee and with the 1964 Helsinki declaration and its later amendments or comparable ethical standards. All applicable international, national, and/or institutional guidelines for the care and use of animals were followed.

### Quantitation of FLNA in human serum samples by ELISA

Antibodies against FLNA were developed by immunizing mice with *E. coli*-expressed partial FLNA protein (aa 1443–2131). Hybridomas were selected by affinity to HEK293-expressed full length FLNA by ELISA and BLI (Bio-layer interferometry). Antibodies were further screened to ascertain no reactivity to Filamin family members FLNB and FLNC. Sandwich ELISA was optimized by R&D Systems using two high affinity FLNA antibodies (3F4 and 6E3 developed by Berg, LLC in-house) and HEK293-expressed full length FLNA as a calibrator.

### Quantitation of FLNB in human serum samples by ELISA

Antibodies against FLNB were developed by immunizing mice with *E. coli*-expressed partial FLNB protein (aa 1416–2089). Hybridomas were selected by affinity to HEK293-expressed full length FLNB by ELISA and BLI (Bio-layer interferometry). Antibodies were further screened to ensure no cross-reactivity to the other two filamin family members, FLNA and FLNC. Sandwich ELISA was optimized by R&D Systems using two high affinity FLNB antibodies (3F10 and 5H7 produced in-house) and HEK293-expressed full length FLNB as calibrator.

### FLNA and FLNB assay validation

The following parameters were assessed during assay validation: Calibration curve precision and accuracy were evaluated using 4-PL non-linear regression model over 6 assay runs. Intra and inter-run QC precision was evaluated over 6 separate daily runs using both lyophilized QC’s and matrix QC’s (n ≥ 34). Short-term stability was evaluated for up to 24 hours at 4°C (FLNA), 6 hours at 4°C, and 4 hours at benchtop (FLNB). Long-term stability was assessed using serum samples stored at −80°C. Spike recovery and dilution linearity (8-fold) were evaluated throughout the assay working range. Freeze-thaw stability of up to three cycles was tested at −80°C. Potential interferences were evaluated by spiking samples with hemoglobin (50 mg/dL), unconjugated bilirubin (3 mg/dL), and triglyceride-rich lipoproteins (2170 mg/dL). Cross-reactivity was evaluated using FLNB recombinant protein on FLNA assay and plate homogeneity was evaluated using spiked matrix.

### Quantitation of KRT19 in human serum samples by ELISA

KRT19 was assessed using a commercially available diagnostic ELISA kit per manufacturer’s instructions (Fujirebio, Malvern, PA). This kit detects the CYFRA-21-1 fragment of KRT19. Manufacturer’s instructions were followed for sample testing.

### KRT19 assay validation

The following parameters were assessed during assay validation: Calibration curve precision and accuracy were evaluated using suggested 4-degree polynomial regression model over 5 assay runs. Intra and inter-run QC precision was verified over 6 separate daily runs using both lyophilized QC’s and serum QC’s (n ≥ 12). Short-term stability was evaluated for up to 24 hours at 4°C, benchtop and 37°C. Freeze-thaw stability was assessed for up to five cycles at −80°C. Spike recovery was evaluated at mid (25 ng/ml) and high (50 ng/ml) analytical range of the assay.

### Quantitation of FLNA peptides by immunoprecipitation and LC-MS/MS (MRM) analysis

#### Antibody immobilization

Three mouse monoclonal antibodies, Anti-FLNA 2C12, Anti-FLNA 3F4, and Anti-FLNA 6E3 (Berg, as used in the ELISA described above) were immobilized onto an agarose support using the ThermoFisher Scientific Pierce Direct IP Kit (ThermoFisher Scientific) according to the manufacturer’s protocol with a few modifications. 200 µg of each of the three antibodies, were coupled individually to 200 µL of AminoLink Plus coupling resin and stored at 4°C until needed.

#### Immunoprecipitation and preparation of calibration standards

Immunoprecipitation was performed using the Pierce Direct IP Kit (ThermoFisher Scientific) according to the manufacturer’s protocol with few modifications. Immunoprecipitation tubes were prepared by aliquoting 5 µL of each of the three antibody-coupled resins into the IP tube (Pierce Direct IP Kit, ThermoFisher Scientific). The resin was washed twice with 200 µL of IP lysis/wash buffer. 100 µL of human serum sample or 100 µL of water (surrogate matrix) was added to each IP tube along with 500 µL of prepared lysis buffer solution (IP lysis/wash buffer with 1.2× Halt protease cocktail inhibitor (ThermoFisher Scientific) and 0.5 M EDTA and incubated overnight at 4°C with end-over-end mixing. The resin was washed five times with 200 µL of IP lysis/wash buffer and once with 100 µL of 1× conditioning buffer. The captured proteins were eluted with 50 µL of elution buffer with an incubation time of 15 minutes and neutralized with 5 µL of 1M Tris HCl, pH 9.0 (Teknova, Hollister, CA). The IP eluates from the surrogate matrix were used to prepare P2 (AGVAPLQV) and P4 (YNEQHVPGSPFTA) peptide calibration curves by spiking with a P2/P4 synthetic peptide (Genscript, Piscataway, NJ) stock solution (0.2/0.36 µg/mL) followed by serial dilution. P2 and P4 calibration standards ranged from 125 pg/mL to 2000 pg/mL and 1125 pg/mL to 36000 pg/mL, respectively. All samples were then subjected to trypsin digestion as described below.

#### Digestion of IP extracted samples using trypsin

Trypsin digestion was performed using the Flash Digest Kit (Perfinity Biosciences, West Lafayette, IN) following the manufacturer’s protocol with few modifications. Flash digest tubes were equilibrated to room temperature and then centrifuged for 1 min at 1500 × g and 5°C. 50 µL of each sample, 25 µL of digestion buffer (Perfinity Biosciences), and 5 µL of working internal standard (ThermoFisher Scientific) solution (P2/P4 10/30 ng/mL) were added to the Flash digest tubes. After vortexing, samples were digested at 70°C for 20 minutes in the Eppendorf, ThermoMixer C (Eppendorf). The Flash digest tubes were then centrifuged for 5 minutes at 1500 × g and 5°C. A 60 µL aliquot of the supernatant was transferred to an LC-MS vial.

#### LC-MS/MS (MRM) analysis

MRM analyses were performed on a 6500 QTRAP mass spectrometer (Sciex) equipped with an electrospray source, a 1290 Infinity UPLC system (Agilent Technologies, Santa Clara, CA) and a XBridge Peptide BEH300 C18 (3.5 µm, 2.1 mm × 150 mm) column (Waters, Milford, MA). Liquid chromatography was carried out at a flow rate of 400 µL/min, and the sample injection volume was 30 µL. The column was maintained at a temperature of 60°C. Mobile phase A consisted of 0.1% formic acid (Sigma Aldrich) in water (ThermoFisher Scientific) and mobile phase B consisted of 0.1% formic acid in acetonitrile (ThermoFisher Scientific). The gradient with respect to %B was as follows: 0 to 1.5 min, 5%; 1.5 to 2 min, 5% to 15%; 2 to 5 min, 15%; 5 to 7.1 min, 15% to 20%; 7.1 to 8.1 min, 20% to 80%; 8.1 to 9.0 min, 80%; and 9.0 to 9.1 min, 80% to 5%. 9.1 to 16 min, 5%. The instrument parameters for 6500 QTRAP mass spectrometer were as follows: Ion spray voltage of 5500 V, curtain gas of 20 psi, collision gas set to “medium”, interface heater temperature of 400°C, nebulizer gas (GS1) of 80 psi and ion source gas (GS2) of 80 psi and unit resolution for both Q1 and Q3 quadrupoles.

#### Selection of surrogate peptides and MRM transitions

Potential surrogate peptides for FLNA quantitation were initially chosen by Skyline software [14] and LC-MS/MS analysis (LTQ Orbitrap Velos coupled to Eksigent nano-LC) of recombinant FLNA protein (GenScript) tryptic digest. From the list of potential surrogate peptides, two surrogate peptides, peptide 2 (AGVAPLQVK) and peptide 4 (YNEQHVPGSPFTAR) were selected based on surrogate peptide selection rules [15] and signal intensities of the peptides in spiked and unspiked serum digests. The uniqueness of the surrogate peptides to the target protein was confirmed by running BLAST searches. Heavy labeled versions of the surrogate peptide 2 and 4, AGVAPLQV[K(13C6; 15N2)] and NEQHVPGSPFTA[R(13C6; 15N4)] were used as internal standards. MRM transitions were optimized using synthetic surrogate peptides (GenScript) and their internal standards (ThermoFisher Scientific) and the following m/z transitions were monitored: P2, 441.7 (M+2H)^2+^→584.5 (y_5_^1+^); P4 535 (M+3H)^3+^→832.4 (y_8_^1+^), P2_IS 445.5 (M+2H)^2+^→592.1(y_5_^1+^); P4_IS, 538.4 (M+3H)^3+^→842.5(y_8_^1+^).

### IPMRM data analysis and quantitation

Data analysis was performed using the Analyst^®^ software (version 1.6.2, AB Sciex, Framingham, MA) and peak integrations were reviewed manually. The calibration curve for FLNA P2 and P4 peptides was constructed by plotting the peak area ratios (analyte/internal standard) versus concentration of the standard with 1/x^2^ linear least square regression. The regression equations from P2 and P4 calibration standards was used to back-calculate the measured P2 and P4 concentrations for each QC and unknown sample.

### Assay validation

The following parameters were assessed during assay validation: Calibration curve linearity and linearity regression weighting factor were assessed from 4 independent calibration curves for P2 and P4. Intra and inter-batch precision of the assay was evaluated by analyzing low QC (LQC) and high QC (HQC) human serum samples (6 replicates each) on different days. LQC and HQC samples were also used to assess sample stability in the autosampler (4°C stored up to 48 hours), short term stability (at 4°C and ambient temperature, stored up to 48 hours), long term stability (at −80°C) and freeze-thaw stability (up to three cycles at −80°C and −20°C), post-preparation stability (at −20°C). Potential interferences were evaluated by spiking HQC and LQC samples with hemoglobin (500 mg/dL), unconjugated bilirubin (30 mg/dL), and triglycerides (1000 mg/dL). In addition, carry over and instrument drift were also assessed.

### Sample collection

Clinically annotated serum samples with PSA values and Gleason scores (GS) were collected from males visiting Mount Sinai Hospital, Toronto, Canada between September 2007 and April 2008 with prostatic symptoms. Samples were collected just before biopsy from 662 patients who underwent prostatic biopsy that resulted in definitive diagnosis of prostatic conditions including prostate cancer (n=311), benign conditions (n=122), atypical small acinar proliferation (n=26), inflammation (n=58), prostatic intraepithelial neoplasia (n=69), microfocus adenocarcinoma (n=16), and benign prostatic hyperplasia (n=60). A total of 503 samples were included in the final analysis; 159 samples were removed from analysis due to missing variable values in either FLNA, FLNB, P2, or P4.

### Statistical analysis

Regression models were built and compared for their ability to classify patients with prostate cancer with low GS (≤6), high GS (≥7), and with an absence of cancer on biopsy. The resulting Prostate Cancer Panel predictive algorithms were based on the regression models and probability threshold values selected to achieve a certain level of test sensitivity or specificity. All analyses were performed in R 3.2.2 with significance level of 0.05, unless otherwise stated.

## Results

This study describes the development of novel biomarker assays to screen for and monitor prostate cancer in concert with the existing PSA test. Two ELISAs were developed for the new biomarkers FLNA and FLNB. The existing commercial assays were found to perform suboptimally. The other biomarker in the panel, KRT19, already had a commercially available ELISA kit for use. Our preliminary investigations on the quantitation of FLNA, FLNB and KRT19 using an MRM-only approach proved to be futile due to insufficient sensitivity. Since our novel biomarkers were low abundance proteins in human plasma/serum matrices, development of quantitative mass spectrometry based assays was particularly challenging. To improve the sensitivity of the assay, an immunoaffinity enrichment approach coupled with MRM was evaluated using the antibodies developed for ELISA. Of the three biomarkers, an IPMRM assay was feasible only for FLNA biomarker in serum matrix.

### FLNA, FLNB and KRT19 ELISA validation

FLNA and FLNB ELISA method development involved optimization of several parameters including antibody pair selection, blocking buffers, assay diluents, incubation times, matrix selection, selectivity and sensitivity. The assay performance was then evaluated by performing a validation study in the serum matrix. Since the KRT19 assay was a commercial kit, validation of the assay was performed in-house. All ELISA assays met validation acceptance criteria as detailed in Table 1.

### FLNA IPMRM assay development and validation

FLNA IPMRM assay development involved optimization of several parameters including: selection of antibodies, immobilization, immunoaffinity capture, incubation, elution, trypsin digestion and other mass spectrometry parameters. The performance of the FLNA IPMRM assay was assessed using serum QCs and the assay met validation acceptance criteria as shown in Supplementary Table 1.

### Comparison of the Prostate Cancer Biomarker Panel FLNA, FLNB and KRT19, versus PSA alone for prediction of prostate cancer

**Figure 1:**
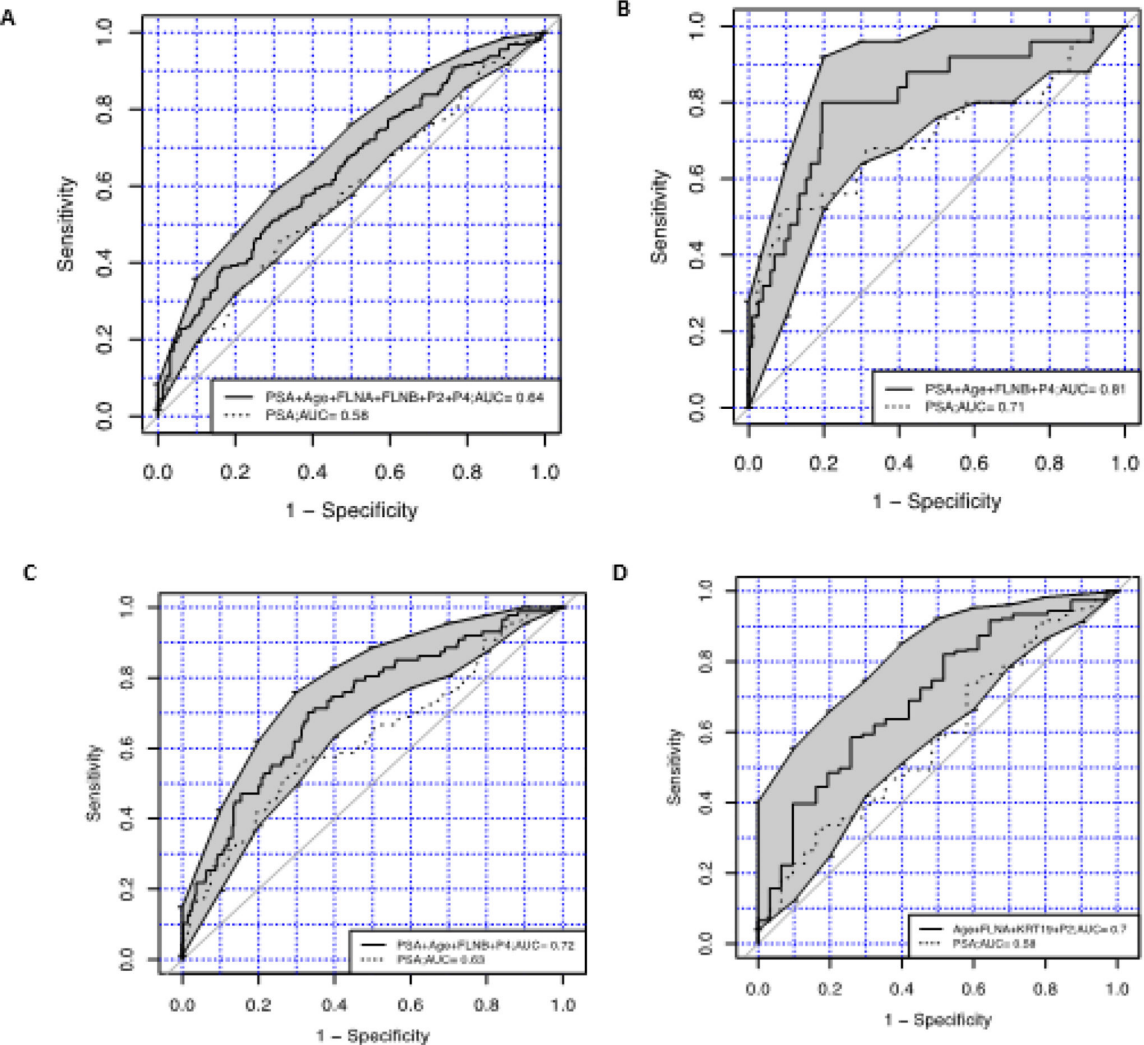
ROC curves of regression models using the prostate biomarker panel, age and PSA test compared to PSA alone **1A**) Prostate Biomarker Panel (FLNA, FLNB, age and PSA) predicts prostate cancer more accurately than PSA alone between patients with or without prostate cancer (Prostate Biomarker Panel AUC, 0.64 (0.59, 0.69), PSA alone AUC, 0.58). **1B**) Prostate biomarker FLNB, Age and PSA discriminates between patients with either Gleason ≤6, or Gleason ≥7, over use of PSA alone (Prostate panel AUC, 0.81, (0.71, 0.9), PSA alone AUC, 0.71). **1C**) Prostate biomarker FLNB, Age, PSA and low Gleason score (≤ 6) predicts likelihood of low-risk disease over use of PSA alone (Prostate panel AUC, 0.72 (0.66, 0.78), PSA alone AUC, 0.63). **1D**) Prostate biomarkers FLNA, KRT19 and Age with PSA discriminates between prostate cancer and benign prostate hyperplasia over use of PSA alone (Prostate panel AUC, 0.71, (0.60, 0.80), PSA alone AUC, 0.58).

**Figure 2:**
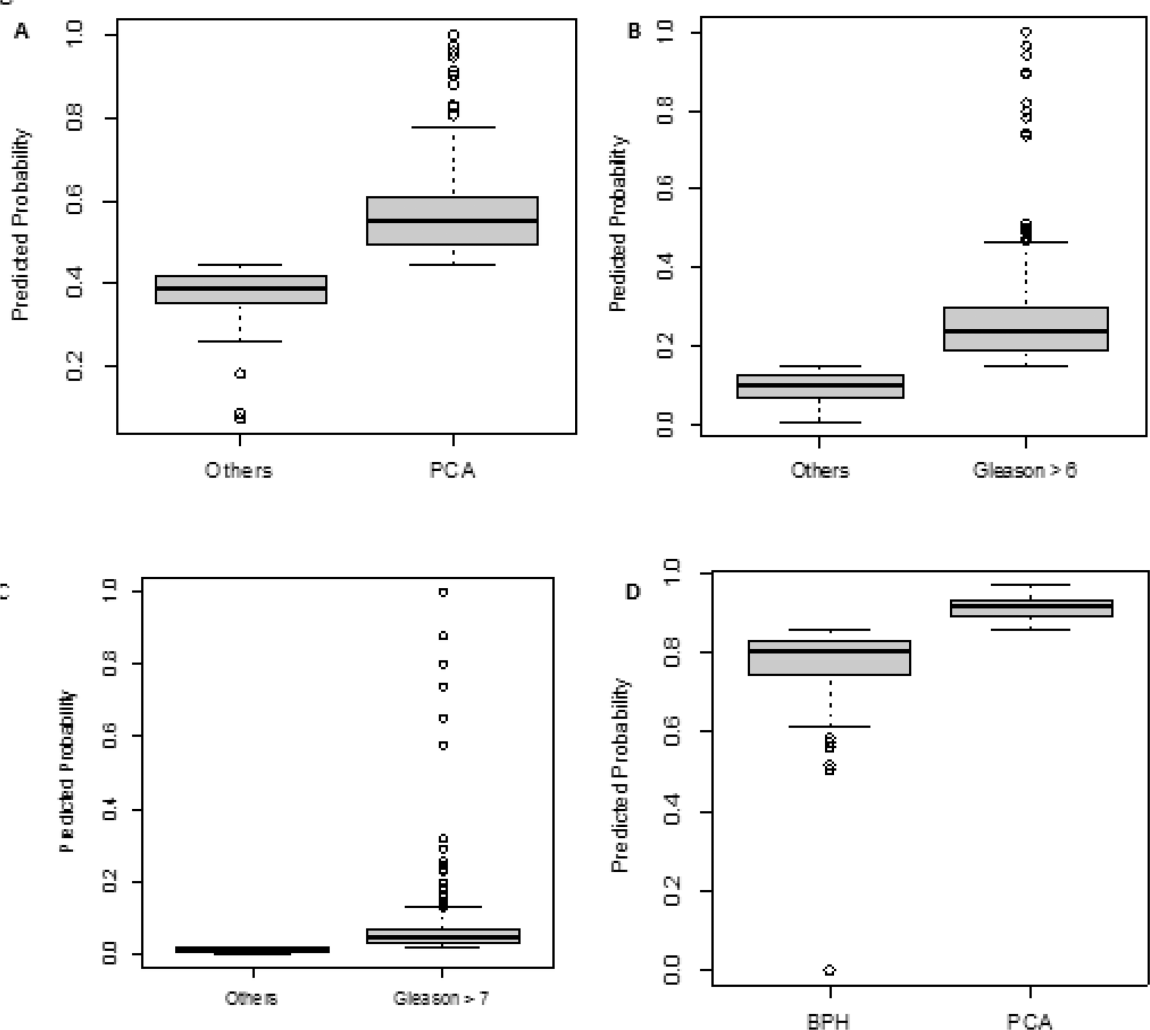
Predicted probability distributions plotted for each ROC analysis **1A**) Distribution of predicted probabilities for patients with or without PrCa. **1B**) Distribution of predicted probabilities for patients with high Gleason score (GS ≥ 7) disease. **1C**) Distribution of predicted probabilities for patients with Gleason score ≤ 6. **1D**) Distribution of predicted probabilities for patients with benign prostatic hyperplasia or PrCa.

Sera from patients were then tested with the biomarker ELISAs and IPMRM. The results were combined with data on age, PSA, and Gleason score and subjected to regression modelling. Table 2 shows the patient demographic data pertaining to age, cancer status, GS and benign classification for the samples analyzed in this study. The Prostate Cancer Biomarker Panel, (biomarkers FLNA, FLNB, Age and PSA) improved the classification of prediction of prostate cancer over PSA alone (AUC=0.64, [0.59, 0.69], vs 0.58) (Figure 1A). The predictive algorithm was set to have a cutoff=0.45, which is based on the regression model achieving sensitivity equivalent to PSA=4 ng/ml. The distribution of predicted probabilities for patients with and without PrCa are shown in Figure 2A.

### High-risk disease prediction with FLNB, age and PSA, compared with PSA alone

Comparing serum samples from patients with high GS (≥7) and samples from patients with low GS (≤6) with our biomarker FNLB, Age and PSA, against the use of PSA alone is shown in Figure 1B. The model that achieved the greatest prediction between patients with GS ≤6 and patients with GS ≥7 is a regression model with the biomarker FLNB, age and PSA. The algorithm was set to have a cutoff=0.02, which is based on the regression model achieving sensitivity ≥0.95. When compared with PSA alone, regression modelling with FLNB, Age and PSA improved the classification of low and high Gleason scores (AUC=0.81 [0.71, 0.90], vs 0.71). The distribution of predicted probabilities for patients with Gleason score ≤6 and Gleason score ≥7 are shown in a box plot (Figure 2B).

### Low-risk disease prediction with FLNA, age and PSA, compared with PSA alone

Patient serum samples with Gleason scores ≤6 were analyzed with our biomarker FLNA, Age and PSA, compared with PSA alone as shown in Figure 1C. The model that achieved the highest prediction for patients with low Gleason score (≤ 6) over PSA alone is a regression model with biomarker FLNB, Age and PSA vs PSA alone. The predictive algorithm was set to have a cutoff=0.15, which is based on the regression model achieving a sensitivity of ≥0.8. The Biomarker Panel FLNB, age and PSA has improved classification over PSA alone (AUC 0.72 [0.66, 0.78] vs 0.63). The distribution of predicted probabilities for patients with low Gleason score are shown in a boxplot in Figure 2C.

### Prediction of benign prostate hyperplasia versus prostate cancer with FLNA, KRT19 and age, compared with PSA alone

Samples of patient sera were analyzed with the biomarkers FLNA, KRT19 and age combined, versus PSA alone. Figure 1D showed the highest prediction between patients with benign prostatic hyperplasia versus PrCa. The predictive algorithm was set to have a cutoff=0.86, which is based on the regression model achieving sensitivity ≥0.8. The biomarkers FLNA, KRT19 and age have improved classification over PSA alone (AUC=0.70 [0.60, 0.80], vs 0.58). The distribution of predicted probabilities for patients with benign prostatic hyperplasia versus PrCa is shown in Figure 2D.

Supplementary Table 2 summarizes the cutoff, AUC, sensitivity, specificity, positive predictive values (ppv), and negative predictive values (npv) of the predictive algorithms for each comparison.

## Discussion

This manuscript describes the validation of a novel Biomarker Panel for prostate cancer screening using traditional ELISA and IPMRM for analysis of patient serum samples. IPMRM combines IP with mass spectrometry and allows the rapid quantitation of proteins with enhanced sensitivity and specificity. For biomarkers, this technique has shown to achieve low ng/mL quantitation by selective enrichment of target proteins in complex matrices [16–18].

Currently, there is an unmet clinical need for a more specific and accurate test for prostate cancer. The standard of care for prostate cancer diagnosis is the PSA test in combination with diagnostic prostate biopsy. However, the PSA test has a high false positive rate and may not reflect true cancer detection. In 2012, the US Preventive Services Task Force issued a recommendation against the use of PSA screening due to the over-detection and overtreatment of non-lethal cancers [3]. To confirm diagnosis, patients undergo invasive prostate biopsies that may cause infection and urinary dysfunction [19].

Continued use of the PSA test and prostate biopsy places a heavy burden on the patient and healthcare system with over-diagnosis, unnecessary biopsies and increased costs. From 2006–2009, Medicare spent $450 million annually on PSA screening and subsequent diagnostic procedures. Additionally, the cost of screening men over 75 years, the population least likely to benefit from the PSA test, was $145 million annually during this time period, representing a third of total Medicare spending on prostate cancer screening [20]. Current efforts focus on the development of non-invasive biomarkers to distinguish between PrCa and benign prostate hyperplasia, aggressive and indolent forms of the disease, with the aim of reducing the number of biopsies performed.

The primary goal of this study was to develop sensitive, specific and reliable assays to quantitate the biomarkers FLNA, FLNB and Keratin-19 in serum and evaluate their clinical utility. The identification of biomarker panels for health problems such as cancer are being used more frequently to address the need to better classify disease groups, predict the effect of therapeutic intervention, and monitor and detect cancer as early as possible [21]. Biomarkers are most often identified as multiple protein panels [22], which then must be verified and validated. Biomarkers can be identified in the low ng/ml range in an MRM multiplex assay, which minimizes assay time and sample volume required [23].

This study addresses the development and clinical validation of a novel biomarker panel for improving the detection of prostate cancer. A recent study by this group discovered three novel biomarkers, FLNA, FLNB and KRT19 for prostate cancer [13,24]. The Prostate Cancer Biomarker proteins in the panel have been shown in previous studies to have links to cancer. Previous work has shown the absence of KRT19 in prostate cancer cells compared to the levels observed in androgen refractory cell lines. This is suggestive of the utility of KRT19 as a biomarker for differentiating aggressive, metastatic forms of PrCa. Additionally, altered levels of KRT19 expression have been demonstrated in the bone marrow of metastatic PrCa patients [25]. FLNA and FLNB belong to a family of large actin-binding filamins and play a major role in cell migration, vascular development, extracellular signaling, and activity of integrins [26]. FLNA has been described previously as being involved in normal prostate physiology and in PrCa metastases [26–28]. FLNB was also shown to be involved in tumor growth and metastases [29].

New assays were developed for FLNA (ELISA and IPMRM) and FLNB (ELISA). The IPMRM assay was especially suited for detection of FLNA in serum, which is more abundant and has been detected in cleaved fragments [30]. ELISA alone may not detect all forms of FLNA in a serum sample. IPMRM allows detection of different peptides along the length of the entire protein. IPMRM assays were not developed for FLNB and KRT19 as both proteins were low in abundance in the serum samples.

In this study, over 500 serum samples were screened against the PrCa Biomarker Panel (FLNA, FLNB, KRT19), and this data was combined with age, PSA test results and Gleason score to assess whether this combinatorial approach was better at predicting prostate cancer, high-risk disease versus indolent disease and discriminating between benign prostate hyperplasia and cancer than the PSA test alone. Data was analyzed by regression modelling. The PrCa biomarkers FLNA, FLNB, age and PSA predicted the likelihood of a patient having prostate cancer better than PSA alone. This was an improvement over the standard PSA test, and could reduce the number of unnecessary biopsies in this population.

The biomarker panel FLNB, age and PSA showed improved sensitivity and specificity over the use of PSA alone in predicting whether patients had Gleason score ≥7 or lower Gleason score ≤6. Additionally, the biomarkers FLNA, KRT19 and age were able to improve the classification of whether patients had benign prostate hyperplasia or cancer over the PSA test alone. Current commercially available tests include the 4K score [31], which has been extensively tested in Europe and the US, and discriminates between high-risk and low-risk disease. This test utilizes the four-kallikrein (KLK) panel immunoassay of KLK2, total PSA, intact PSA, and free PSA in combination with a patient’s age, DRE results and prior biopsy status. This information is analyzed by an algorithm to determine the percentage risk for aggressive prostate cancer. 4Kscore test was used in a prospective validation study in the US in 2014, and performed well in identifying patients with high risk disease. However, this test still requires a prostate biopsy and is heavily dependent on PSA levels. In comparison, the test described in this manuscript with FLNA, FLNB and KRT19, does not require invasive procedures to be performed on patients, and is also able to distinguish between benign prostate hyperplasia and prostate cancer. The PHI (prostate health index) test also discriminates between high and low risk cancers. PHI was shown in a European study to be more accurate than PSA alone in predicting prostate cancer in obese patients [32]. However, it is unclear at present if PHI can discriminate between intermediate PSA values [33–35].

Prostate cancer antigen 3 (PCA3) is a non-PSA-based test of the expression of long non-coding RNA that is elevated in over 90% of PrCa tissue, but is not found in BPH or healthy tissues [36]. This is a non-invasive urine test that in combination with PSA improves PrCa prediction [37]. The androgen-induced transmembrane protease, serine 2 (*TMPRSS2-ERG*) is detected in urine samples of suspected PrCa patients. However, *TMPRSS2-ERG* is absent in 50% of cancers, and therefore it must be multiplexed with other biomarkers such as PCA3 [38]. In a study of 1300 men combination testing with *TMPRSS2-ERG* and PCA3 improved the sensitivity of PrCa diagnosis [39]. However, both PCA3 and *TMPRSS2-ERG* are dependent on relative PSA expression diagnosis [39,38]. The biomarker alpha-methylacyl-CoA racemase (*AMACR*), detected by RNA expression profiling demonstrates high sensitivity and specificity in prostate biopsy tissue [40]. However this biomarker is not specific to prostate cancer, nor can it be used for detection of invasive cancer in urine [41], but can be used when prostate biopsy analysis is ambiguous [42]. In conclusion, the Prostate Cancer Biomarker Panel developed in this study demonstrates an advantage over existing tests in that it not only discriminates between high and low-risk disease, it also discriminates between cancer and benign prostate hyperplasia. Use of these biomarkers will potentially allow for more accurate diagnostic and treatment decisions, and improve the accuracy of disease prognosis by better distinguishing between indolent and high-risk disease.

## Supplementary Material

Supplemental Tables

## Figures and Tables

**Table 1:** FLNA, FLNB and KRT19 ELISA validation summary. All levels reported in this table are within the appropriate acceptance criteria.

Study	FLNA	FLNB	KRT19
Analytical Range	3.13 ng/ml to 200 ng/ml	0.087 ng/ml to 2.79 ng/ml	0.5 ng/ml to 50 ng/ml
R^2^ of calibration curves	≥ 0.99	≥ 0.99	≥ 0.99
Intra-day Precision	CV<10% (n=8)	CV<14.6% (n=5)	CV<6.7% (n=8)
Inter-day Precision	CV<8.7% (n=41)	CV<23% (n=34)	CV<19.2% (n=15)
Spike Recovery in serum	124.20%	89%	98–121%
Dilutional Linearity in serum	%bias <20% for up to 1:8 dilution	%bias <20% for up to 1:8 dilution	N/A
Freeze-Thaw Stability in serum	Stable up to 3 freeze-thaw cycles	Stable up to 3 freeze-thaw cycles	Stable up to 5 freeze-thaw cycles
Short-term Stability in serum	Stable for 2 hours at room temperature and at 6 hours at 4°C	Stable for 4 hours at room temperature and 6 hours at 4°C	Stable for 4 hours at room temperature and 24 hours at 4°C
Long-term Stability in serum	Stable for up to 1 year at −80°C	Stable for up to 1 year at −80°C	N/A
Interfering Substances in serum	No interference for levels below 250 mg/dL Hemoglobin; 30 mg/dL Bilirubin; 1000 mg/dL Lipoproteins	No interference for levels below 50 mg/dL Hemoglobin; 3 mg/dL Bilirubin; 2170 mg/dL Lipoproteins	N/A
Specificity in serum	No cross reactivity with FLNB protein at 10 pM	No cross reactivity with FLNA protein at 179 pM	N/A

**Table 2:** Patient demographic data for the samples in this study. Table shows number of benign and cancer cases, Gleason scores and benign classification breakdown for the population studied.

	Age Range	Mean	SD
BPH	48–75	59	11
Benign	45–82	61	7
Gleason ≤6	42–84	62	7
Gleason =7	45–82	65	8
Gleason >7	52–83	68	7
**Cancer Classification**			
Benign	224		
Cancer	279		
**Total**	**503**		
**Gleason Score**			
<6	2		
6	156		
7	74		
≥8	28		
Gleason score N/A	243		
**Total**	**503**		
**Benign Classification**			
Benign	81		
N/A	6		
Other-Benign Prostate Hyperplasia	34		
Other-Inflammation	46		
Other-Prostatic Intraepithelial Neoplasia	57		
**Total**	**224**		

Note: Patient demographic data for the samples in this study. Table shows number of benign and cancer cases, Gleason scores and benign classification breakdown for the population studied.

## Abbreviations

PSA: Prostate Specific Antigen
GS: Gleason Score
FLNA: Filamin-A
FLNB: Filamin-B
KRT19: Keratin-19
IPMRM: Immunoprecipitation Multiple Reaction Monitoring
