# Supplementary material for: Clinical Validation of a Serum Protein Panel (FLNA, FLNB and KRT19) for Diagnosis of Prostate Cancer
Source: J Mol Biomark Diagn. Author manuscript; Available in PMC 2018 Apr 20. (PMC5909981; doi:10.4172/2155-9929.1000323)
Supplement: Supplemental Tables [file NIHMS905432-supplement-Supplemental_Tables.docx]

| **Study** | **FLNA IP-MRM P2** | **FLNA IP-MRM P4** |
| --- | --- | --- |
| Analytical Range | 125 pg/mL – 2000 pg/mL | 1125 pg/mL – 36000 pg/mL |
| R^2^ of calibration curves | ≥0.99 | ≥0.99 |
| Intra-day Precision | LQC CV≤23% (n=6)  HQC CV≤11% (n=6) | LQC CV≤13% (n=6)  HQC CV≤10% (n=6) |
| Inter-day Precision | LQC CV=13% (n=36)  HQC CV=18% (n=36) | LQC CV=30% (n=36)  HQC CV=14% (n=36) |
| Selectivity (Surrogate Matrix) | Interference ≤19%, IS ≤4% | No interference |
| Weighting Factor | 1/x^2^ | 1/x^2^ |
| Linearity (Calibration standards) | % bias ≤5% | % bias ≤9% |
| Carryover (Analyte/IS) | None | None |
| Autosampler Stability | Samples stable up to 48 hr at 4°C | Samples stable up to 48 hours at 4°C |
| Interference:  Hemoglobin 500 mg/dL  Bilirubin 30 mg/dL  Lipids 1000 mg/dL | Interference not significant | Interference not significant |
| Short Term Stability inSerum | 24hr benchtop and 4°C stability | 24hr benchtop and 4°C stability |
| Long Term Stability in Serum | Up to 11 months | Up to 5 months |
| Post-Preparative Stability | Extracted samples stable up to 48hr at -20°C | Extracted samples stable up to 48hr at -20°C |
| Freeze-Thaw Stability | Samples are stable up to 3 freeze-thaw cycles | Samples are stable up to 3 freeze-thaw cycles |
| Short-Term Stock Solution Stability  (on ice for 6hr) | Peptide solutions stable on ice for 6hr  IS solutions will be prepared immediately before use | Peptide solutions stable on ice for 6hr  IS solutions will be prepared immediately before use |

**Supplemental Table 1.**

**Supplemental Table 2.**

| **Model** | **Cut off** | **AUC** | **Sensitivity** | **Specificity** | **PPV** | **NPV** | **OR** |
| --- | --- | --- | --- | --- | --- | --- | --- |
| **PCA vs Other** | **0.45** | **0.64** | **0.74** | **0.43** | **0.57** | **0.62** | **2.16 (1.45, 3.21)^1^** |
| **Gleason >=8 vs Other** | **0.02** | **0.81** | **0.96** | **0.25** | **0.07** | **0.99** | **8.05 (1.08, 60.22)^1^** |
| **Gleason >=7 vs Other** | **0.15** | **0.72** | **0.80** | **0.52** | **0.29** | **0.92** | **4.45 (2.52, 7.85)^1^** |
| **PCA vs BPH** | **0.86** | **0.70** | **0.80** | **0.48** | **0.92** | **0.25** | **3.83 (1.76, 8.33)^1^** |

^1^ Significance level<0.05
